# Cardiorespiratory and oxygenation responses in iron-deficient anemic women during whole-body exercise under moderate hypoxia

**DOI:** 10.1007/s00421-025-05940-w

**Published:** 2025-09-01

**Authors:** Panagiotis G. Miliotis, Spyridoula D. Ntalapera, Panagiotis Lakeas, Ioannis Loukas, Argyris G. Toubekis, Nickos D. Geladas, Maria D. Koskolou

**Affiliations:** 1 Division of Sport Medicine and Biology of Exercise, School of Physical Education and Sport Science, National and Kapodistrian University of Athens, Athens, Greece; 2 Division of Aquatic Sport, School of Physical Education and Sport Science, National and Kapodistrian University of Athens, Athens, Greece; 3 Division of Sciences, School of Physical Education, Sport and Exercise Sciences, University of Otago, Dunedin, New Zealand

**Keywords:** Iron deficiency anemia, Muscle and cerebral oxygenation, Hypoxemia, Whole-body exercise, Exercise tolerance, Cardiovascular responses

## Abstract

**Purpose:**

Integrated physiological responses during maximal whole-body exercise, such as cycling, under additive hypoxemia (anemia + hypoxia) are not adequately studied. Therefore, we investigated cardiovascular, muscular and cerebral oxygenation responses in chronic mildly iron-deficient and control women under normoxic and moderate hypoxic conditions during maximal whole-body exercise.

**Methods:**

In a randomized and counterbalanced order, 16 females performed incremental exercise to exhaustion under normoxia (N; FIO_2_:20.94%) and hypoxia (H; FIO_2_:13.6%). The participants were divided into two groups matched for age and anthropometric characteristics, but intentionally varying in [Hb] (*p* < 0.001) and V̇O_2max_ (*p* < 0.01); iron-deficient (A; *n* = 8; [Hb]:11.3 ± 0.4 g/dl; V̇O_2max_:37.3 ± 2.8 ml/kg/min) and healthy controls (C; *n* = 8; [Hb]:13.3 ± 0.4 g/dl; V̇O_2max_:40.8 ± 1.9 ml/kg/min).

**Results:**

During exercise in hypoxia compared to normoxia, the A exhibited greater decrement in V̇O_2max_ (5.0%; *p* = 0.02) and peak power output (5.4%; *p* = 0.004) than C. Maximal mean arterial pressure was reduced (*p* < 0.05) due to lower total peripheral resistance (*p* < 0.05) and unchanged maximal cardiac output (*p* > 0.05). Enhanced O_2_ utilization under H was observed only in C, based on ΔHHb (*p* < 0.05). Cerebral oxygenation was reduced linearly with CaO_2_ (*r* = 0.95, *p* < 0.001).

**Conclusion:**

Collectively, moderate hypoxia induced greater reduction of V̇O_2max_, peak power output and cerebral oxygenation leading to exercise intolerance in A compared to C. These responses were accompanied by an inability of skeletal muscle to increase O_2_ utilization at maximal effort in H and by a failure of the cardiovascular system to compensate and counteract convective and diffusion limitations during maximal whole-body exercise in anemic women.

## Introduction

Cumulative evidence supports that V̇O_2max_ is limited primarily by oxygen delivery (DO_2_) [cardiac output (*Q*) × arterial oxygen content (C_a_O_2_)] in healthy adults (Saltin and Calbet 2006; Lundby et al. 2017; Ferretti 2014). Reduction of C_a_O_2_ by manipulating hemoglobin concentration [Hb] or FIO_2_ induces peripheral and/or central fatigue and a proportional decrease in V̇O_2max_ (Amann et al. 2006a, 2006b).

During submaximal exercise, in conditions with reduced C_a_O_2_, such as acute isovolemic anemia, carbon monoxide inhalation or hypoxia, the diminished DO_2_ is compensated for by increased limb blood flow, *Q* and vascular conductance (Rowell et al. 1986; Koskolou et al. 1997b, 1997a; Richardson and Guyton 1959; Gonzalez-Alonso et al. 2001; Calbet et al. 2003, 2002). However, at peak exercise, Q does not further increase, leading to diminished exercise tolerance due to lower DO_2_, even when a small muscle mass is engaged (Koskolou et al. 1997b). In cases of chronic severe anemia, systemic and vascular adaptations occur to partially counteract the detrimental effects of reduced [Hb] on DO_2_, thus reaching maximal *Q* levels at low peak power outputs (Sproule et al. 1960).

The co-existence of anemia and hypoxia as a hypoxemic model offers the opportunity to separate the effects of C_a_O_2_ or P_a_O_2_ in cardiorespiratory, muscular and cerebral responses. However, this innovative model is not amply studied. A study with matched C_a_O_2_ but different P_a_O_2_ (hypoxia and anemia) and with matched P_a_O_2_ but different C_a_O_2_ (hypoxia and anemia + hypoxia) showed that the regulation of *Q*, limb blood flow and O_2_ delivery is mainly related to C_a_O_2_ and not to P_a_O_2_ during two-legged knee-extension exercise (Roach et al. 1999). Additionally, a recent study showed that the hypoxic reduction of V̇O_2max_ during cycling exercise was exaggerated in an anemic group compared to control, but there were no available data regarding blood pressure and both muscle and cerebral oxygenation. (Koskolou et al. 2023). Interestingly, no other study has reported cardiovascular responses during maximal whole-body exercise, in humans, under additive hypoxemia (anemia + hypoxia). In fact, the previous studies that applied the current experimental approach of hypoxemia either used smaller muscle mass exercise (2-leg extension) or they did not record MAP and *Q*.

During knee-extension exercise with either one or two legs, maximal cardiac output is lower than during exercise involving larger muscle mass, such as cycling, even in hypoxic conditions (Saltin et al. 1998; Roach et al. 1999; Calbet et al. 2009; Andersen and Saltin 1985) setting a crucial question regarding blood flow redistribution among active and inactive tissues. Additionally, it would be of interest to examine whether oxygen extraction, especially at peak exercise, apart from the small oxygen gradient due to reduced P_a_O_2_ in hypoxia, could be further constrained by iron-deficiency anemia and the corresponding low ferritin values, since iron containing enzymes are involved in oxygen extraction. In severe hypoxemia (C_a_O_2_: 14 ml/dl), the reduced brain oxygenation can cause central fatigue through corticospinal inhibition of motor drive resulting in earlier termination of exercise and V̇O_2max_ reduction (Millet et al. 2012; Vogiatzis et al. 2011; Goodall et al. 2012). Thus, the scenario of additive hypoxemia presents a unique challenge as it may be highly demanding for arterial blood pressure regulation and potentially impair tissue oxygenation to a great extent consequently affecting exercise tolerance.

Therefore, it was hypothesized that anemic individuals with iron deficiency would suffer greater reduction in V̇O_2max_ and cerebral oxygenation compared to controls, during cycling exercise under hypoxia. It was also hypothesized that the anemic group would exhibit higher skeletal muscle vasodilation in hypoxia, as both hypoxia and anemia are known to stimulate compensatory mechanisms through ATP and NO, that promote vasodilation Dinenno 2016; Ellsworth et al. 1995). As a result, arterial blood pressure at maximal exercise would be lower compared to controls. Thus, the aim of the present study was to investigate the cardiorespiratory, muscular and cerebral oxygenation responses of young women with chronic mild iron-deficiency anemia during whole-body exercise under normoxia and hypoxia (FIO_2_: ~ 14%).

## Methods

Sixteen young adult women, eight with chronic mild iron-deficiency anemia (IDA) and eight controls (C) with normal [Hb] volunteered for the study. Based on previous work in our lab (Koskolou et al. 2023) and assuming a power of 0.80 and a statistical significance of *p* < 0.05, at least eight participants were needed for each group (GPower 3.1.9.6, Heinrich-Heine-Universität, Düsseldorf). Both groups had no previous history of cardiovascular or pulmonary diseases and were free of musculoskeletal injuries and unacclimatized to altitude throughout the experiments. Also, all participants had regular menstrual cycle (A: 28 ± 1.4 days and C: 28 ± 0.5) and used no contraception for at least 6 months before experiments. The two groups were matched for age, height, body mass, and %fat. Moreover, the two groups were matched for endurance training level using the prediction equation of differences in V̇O_2max_ with corresponding differences in hemoglobin concentration (Calbet et al. 2006). More specifically, V̇O_2max_ and [Hb] values of the anemic participants were measured, and then, control participants were matched with the anemic based on [Hb]-corrected V̇O_2max_ values.

### Study design

Prior to the main experiments, familiarization with experimental procedures and the equipment was conducted along with a baseline blood status assessment. Thereafter, all participants visited the lab on two separate days to exercise under either normoxia (FIO_2_: 20.94%) (Anemic Normoxia: AN; Control Normoxia: CN) or hypoxia (FIO_2_: 13.6%) (Anemic Hypoxia: AH, Control Hypoxia: CH), in a randomized and counterbalanced order in a similar way for both groups, with at least 48-h interval, during follicular phase assessed with urine test sticks. All participants avoided exhaustive exercise and caffeine consumption for at least 48 h and 12 h before experiments, respectively. The day before the first experiment the participants were asked to record their diet and follow a certain hydration protocol provided by the researchers, to repeat the same food and drink consumption the day prior to the second visit. During experiments, the participants were blind with respect to FIO_2_; they sat on the cycle ergometer for 5 min in normoxic conditions while breathing through a two-way valve (Hans-Rudolph), and thereafter, they were connected to a hose attached to a Douglas bag containing either hypoxic gas (hypoxic condition: H) until SpO_2_ reached < 95% or room air (normoxic condition: N) for 5 min, followed by a 5-min warm up period at 50 W. The exercise load was initially set at 20 W and increased by 20 W every min until volitional exhaustion. The highest 20-s values were averaged to determine V̇O_2max_ (Martin-Rincon and Calbet 2020). Exhaustion was considered when at least three of the following criteria were met (1) an increase in oxygen uptake (VO_2_) less than 150 ml min^−1^, (2) heart rate ≥ 90% HRmax based on the age-predicted maximum, (3) respiratory exchange ratio (RER) ≥ 1.10, (4) rate of perceived exertion (RPE) ≥ 17, and (5) inability to sustain the pedal rate > 50 rpm. The peak power output (PPO) was calculated based on the equation *W*_max_ = *W*_comp_ + *t*/60 × Δ*W *(Kuipers et al. 1985).

### Cardiovascular parameters

The cardiovascular variables: systolic (SP), diastolic (DP), mean arterial blood pressure (MAP), stroke volume (SV), Q, and total peripheral resistance (TPR) were continuously recorded noninvasively via a photoplethysmograph with the cuff attached on the middle finger of the left hand (Finometer 2003, FMS, The Netherlands). This method has been shown to have very high validity correlation (0.93–0.99) with invasive methods such as arterial line placement (Gallagher et al. 2001). Before each experiment, Finometer device was automatically calibrated for pressure, distance of finger sensor from the heart level and detection of sound derived from “return to arm flow”, according to the manufacturer's standards. To minimize measurement’s noise, the left hand was carefully and comfortably immobilized in a stable structure so as not to constrain maximal effort during exercise. Heart rate (HR) was monitored with a standard lead II electrocardiogram (ECG 100C, BIOPAC Inc., USA).

### Muscle and cerebral oxygenation

Both muscle and cerebral oxygenation were monitored online using a high-resolution near-infrared spectroscopy (NIRS) system (Portamon & Portalite, Artinis Medical Systems, The Netherlands) at a dual wavelength (760 nm and 850 nm) of emitting light and three light source transmitters at 30-, 35-, and 40-mm distance to the receiver. NIRS signal was recorded throughout the experiment at a frequency of 10 Hz and the last 20 s of each exercise stage were averaged. A standard differential path length factor (DPF) of 4.0 for the vastus lateralis and 6.0 for the left prefrontal cortex was selected. To determine local muscle oxygenation profiles, the NIRS emitter and detector pair were attached to the middle portion of the left vastus lateralis muscle at the mid-thigh level and parallel to the long axis of the muscle (~ 15 cm above the proximal border of the patella and ~ 5 cm lateral to the midline of the thigh). A Harpenden skinfold caliper was used to measure skin and adipose tissue thickness at the area of the NIRS probe on the VL. Cerebral oxygenation was assessed with an NIRS probe placed on the forehead over the left prefrontal cortex. The NIRS optode was placed 2 cm above the left eyebrow and positioned as laterally as possible from the longitudinal cerebral fissure, targeting the left prefrontal cortex region corresponding approximately to the Fp1 site of the 10–20 EEG system. The NIRS optodes were held in place by a flexible, plastic spacer with fixed optode distance of 4.5 cm and secured on the skin (shaved and cleaned) and the scalp using double-sided waterproof adhesive tape. Cerebral NIRS was recorded at V̇O_2max_. For muscle derived measurements, prior to experimental protocols, a “physiological calibration” was conducted by inflating thigh cuffs at 300 mmHg until HHb (deoxygenated hemoglobin) reached a plateau. Afterward, the average of the highest ΔΗΗb (change in deoxygenated hemoglobin) values sustained for 20 s at 100% of V̇O_2max_ was determined. This average was then expressed as a percentage of the maximum values obtained during muscle ischemia. ΔHHb can be used as an estimate of skeletal muscle fractional O_2_ extraction which is relatively insensitive to blood volume changes (Grassi and Quaresima 2016). For cerebral derived measurements, the absolute values of Tissue Saturation Index (TSI %), and changes (*Δ*) of O_2_Hb (oxygenated hemoglobin), HHb and tHb (total hemoglobin) were used for further analysis.

### Respiratory variables

Gas exchange and ventilatory variables [pulmonary ventilation (V̇_E_), respiratory rate (RR), tidal volume (V_T_), respiratory equation (RER), ventilatory equivalents for oxygen (V̇_E_/V̇O_2_) and carbon dioxide (V̇_E_/V̇CO_2_), and end-tidal oxygen (P_ET_O_2_) and carbon dioxide (P_ET_CO_2_) tensions] were recorded continuously throughout experiments breath by breath via open-circuit spirometry (Ultima CPX, MedGraphics, USA). Before each test, the gas analyzers for O_2_ and CO_2_ and the pneumotachograph were calibrated with two different gas mixtures a) 12% O_2_ and 5% CO_2_ balanced in N_2_ and b) 21% O_2_ and 0.01% CO_2_ balanced in N_2_ and a 3-L syringe (Ultima CPX, MedGraphics, USA), respectively.

### Blood analysis

Hct and [Hb] measurements were taken before exercise during the normoxic period and immediately after exhaustion in duplicate samples. The Hct values were determined using Hct scale (Haematocrit Reader, Hawksley Inc., UK) after a 5-min centrifugation of 75 μL capillary blood at 11,500 rpm (Micro Haematocrit Mk5 Centrifuge, Hawksley, UK) with a resolution of 1% units. The [Hb] values were determined by a portable photometer (Diaspect Tm, Germany) using 10 μL capillary blood. Peripheral oxygen saturation (SpO_2_) was monitored continuously using a finger pulse oximeter (Nellcor Symphony, N-3000, USA) and was recorded throughout the experiment. CaO_2_ was calculated as CaO_2_ = SpO_2_ × [Hb] × 1.34.

### Statistical analysis

After checking the data for normality with Shapiro–Wilk test, a two-way ANOVA (group x FIO_2_) was conducted for cardiovascular, respiratory and tissue oxygenation variables. In case of significant main effect or interaction, a Tukey post hoc test was performed for further comparisons. A two-tailed *t *test for independent samples was conducted for detecting differences between groups in resting baseline characteristics and for Δ hypoxia-derived changes of V̇O_2max_ and PPO. A regression analysis was conducted for mean values of C_a_O_2_ and TSI% at the end of exercise. The level of significance was set at *p* ≤ 0.05. The data are presented as means ± standard deviations. The statistical analysis software used was Statistica v8.

## Results

*Hematological parameters*: The two groups differed by design in baseline [Hb] by ~ 15%, as well as in Hct and ferritin values (Table 1). When the control group was exposed to hypoxia, the C_a_O_2_ levels were similar (*p* > 0.05) to those observed in the anemic group under normoxia and the lowest level of C_a_O_2_ was attained in the anemia + hypoxia condition (*p* < 0.01) (C: 17.6 ± 0.4 ml/dl CH: 15.3 ± 0.7 ml/dl, A: 14.9 ± 0.5 ml/dl, AH: 12.9 ± 0.9 ml/dl).

**Table 1:** Participants’ (*n* = 16) traits (mean ± sd) divided into control and anemic group

	Control (*n* = 8)	Anemia (*n* = 8**)**
Height (cm)	166.5 ± 2.6	164.1 ± 2.4
Body weight (kg)	56.4 ± 4.5	57.3 ± 4.5
Age (yrs)	22.4 ± 2.1	22.6 ± 4.3
FAT (%)	18.9 ± 5.7	20.6 ± 5.5
Hct (%)	40.4 ± 1.1	34.1 ± 1.2***
[Hb] (g/dl)	13.3 ± 0.4	11.3 ± 0.4***
Ferritin (ng/ml)	21.8 ± 5.5	6.9 ± 3.2***
V̇O_2max_ (ml/kg/min)	40.8 ± 1.9	37.3 ± 2.8**

*FAT* body fat percentage, *Hct* hematocrit, *[Hb]* hemoglobin concentration, *V̇O*_*2max*_ maximal oxygen uptake

^**^p < 0.01, ***p < 0.001 differences between groups

### Exercise capacity

**Fig. 1:**
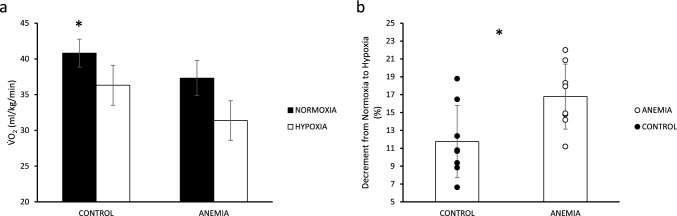
**a** V̇O_2max_ values (mean ± sd) for the anemic (*n* = 8) and the control (*n* = 8) group in normoxia (filled bars) and hypoxia (open bars). *:*p* < 0.05 difference between groups in the same condition. **b** Percent V̇O_2max_ decrement from normoxia to hypoxia (individual values and mean ± sd) for the anemic and the control group. *:*p* < 0.05 difference between groups

Exhaustion criteria during incremental exercise were met for both groups and conditions according to RPE (AN: 17.6 ± 0.7, ΑΗ: 18.0 ± 1.2 vs. CN:17.9 ± 0.8, CH:18.0 ± 0.9) (*p* > 0.05) and RER values (Table 2) and HRmax (see results of cardiovascular variables). Normoxic V̇O_2max_ was different between the two groups by ~ 9% (A: 37.3 ± 2.8 ml/kg/min and C: 40.8 ± 1.9 ml/kg/min, *p* < 0.01). Hypoxia elicited reductions (*p* < 0.001) in V̇O_2max_ in both groups (Fig. 1a), with the percentage drop being greater in the A group (Fig. 1b) compared to controls (A: 16.8 ± 3.6% vs. C: 11.8 ± 4.0%, *p* = 0.02). Peak power output (PPO) was not significantly different between the two groups in normoxia (A: 176 ± 22 W and C: 188 ± 14 W, *p* = 0.2). Hypoxia reduced PPO in both groups; however, the anemic women suffered a greater percentage reduction of PPO in hypoxia compared to controls (A: 13.1 ± 2.4% vs. C: 7.7 ± 3.8%, *p* < 0.01).

**Table 2:** Ventilatory response at maximal effort (mean ± sd) in the control (n = 8) and anemic (n = 8) group under normoxia (Norm) and hypoxia (Hypo)

	Control (*n* = 8)		Anemia (*n* = 8)	
	Norm	Hypo	Norm	Hypo
V̇_E_ (L/min)	103 ± 10	105 ± 11	98 ± 12	101 ± 10
Rf (br/min)	60 ± 8	61 ± 9	57 ± 7	59 ± 7
Vt (ml)	1692 ± 183	1752 ± 239	17,201 ± 229	1724 ± 219
P_ET_CO_2_ (mmHg)	33.8 ± 3.7	31.5 ± 5.2	34.7 ± 3.5	30.1 ± 3.0^a^**
P_ET_O_2_ (mmHg)	114 ± 4	71 ± 4***	116 ± 4	74 ± 4***
RER	1.28 ± 0.09	1.31 ± 0.10	1.30 ± 0.07	1.35 ± 0.06
V̇_E_/V̇O_2_	43 ± 5	48 ± 2	45 ± 4	52 ± 3***
V̇_E_/V̇CO_2_	35 ± 3	36 ± 3	36 ± 2	39 ± 3^a^*
SpΟ_2_	99 ± 1	85 ± 3***	99 ± 1	85 ± 4***

*V̇*_*E*_ minute ventilation, *Rf* respiratory frequency, *Vt* tidal volume, *P*_*ET*_*CO*_*2*_ end-tidal pressure of carbon dioxide, *P*_*ET*_*O*_*2*_ end-tidal pressure of oxygen, *RER* respiratory exchange ratio, *V̇*_*E*_*/V̇O*_*2*_ ventilatory equivalent for oxygen, *V̇*_*E*_*/V̇CO*_*2*_ ventilatory equivalent for carbon dioxide *SpO*_*2*_: oxygen saturation

^*^:*p* < 0.05, **:*p* < 0.01, ***:*p* < 0.001 different from normoxia

^a^:*p* < 0.05 significant difference between groups for the same condition

### Respiratory variables

According to the analysis of mean maximal values for the respiratory variables (Table 2), V̇_E_ and RER did not differ between groups and conditions, whereas *V̇*_E_/V̇O_2_ and *V̇*_E_/V̇CO_2_ were increased (*p* < 0.001 and *p* = 0.02, respectively) and P_ET_CO_2_ was decreased (*p* < 0.01) when transitioning from normoxia to hypoxia only in the A group. Also, a significant decrement (*p* < 0.001) of the same magnitude for the two groups was recorded for P_ET_O_2_ from normoxia to hypoxia. Moreover, SpO_2_ was significantly reduced in hypoxia compared to normoxia, to a similar extent in both groups (Table 2).

### Cardiovascular variables

**Fig. 2:**
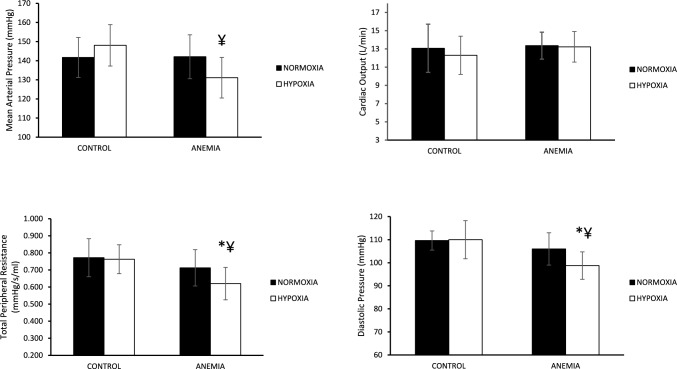
**a** Mean ± sd values for cardiovascular variables under normoxic (filled bars) and hypoxic (open bars) conditions between control and iron-deficient anemic group, at maximal effort. *:*p* < 0.05 between groups in the same condition (anemia vs control). ¥: p < 0.05 different from normoxia in the same group

Similar maximal heart rate responses were recorded across conditions in the two groups (AN: 185 ± 7 bpm AH: 185 ± 9 bpm and CN: 184 ± 6 bpm CH: 183 ± 4 bpm, *p* > 0.05), indicating similar level of exhaustion. Similarly, no difference was noticed in stroke volume and, consequently, in maximal cardiac output (Fig. 2) between the two groups either in normoxia or in hypoxia (p > 0.05).

At maximal exercise level, ANOVA analysis revealed significant interaction for mean arterial pressure response (*p* = 0.03) (Fig. 2), as it was lower in hypoxia compared to normoxia only in the anemic group (*p* = 0.02). Similarly to MAP, an interaction was observed in TPR (*p* = 0.001) and group main effect for DP (*p* = 0.01); in the A group, TPR and DP hypoxic values were lower than normoxic (p < 0.05) and, also, when compared to hypoxic values of the C group (*p* < 0.05) (Fig. 2), while SP was not significantly different between groups and conditions (AN: 199 ± 17 mmHg, ΑΗ: 197 ± 19 mmHg vs. CN: 193 ± 12 mmHg, CH:202 ± 14 mmHg) (*p* > 0.05).

### Tissue oxygenation variables

**Fig. 3:**
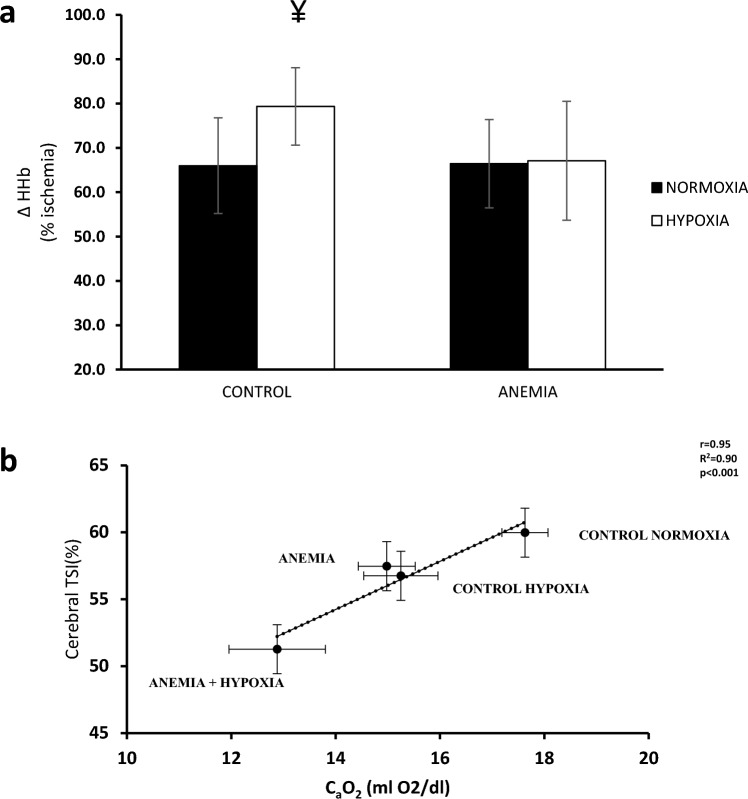
**a** Mean ± sd values for change in muscle deoxygenated hemoglobin (HHb), expressed as % of muscle ischemia, under normoxic (filled bars) and hypoxic (open bars) conditions between control group and iron-deficient anemic group, at maximal effort. ¥: *p* < 0.05 different from normoxia in the same group. **b** Linear relationship between the cerebral tissue saturation index (TSI%) and arterial oxygen content (C_a_O_2_). Each point represents the mean value of each group and condition, at maximal effort

Regarding muscle oxygenation, ΔHHb expressed as % of muscle ischemia showed a significant interaction (*p* < 0.001) (AN: 66.4 ± 10%, AΗ: 67.1 ± 13.4% vs. CN: 66.0 ± 10.8%, CH: 79.4 ± 8.7%); the C group exhibited increased ΔHHb values when transitioning from normoxia to hypoxia (*p* < 0.001), whereas the A group did not show different response in the two conditions (Fig. 3a). No statistical significance between groups or conditions was found either for ΔΟ_2_Hb or ΔtHb (*p* > 0.05).

Regarding cerebral oxygenation, ΔHbO_2_ was decreased in hypoxia (*p* < 0.001) and there was a trend for group × condition interaction (*p* = 0.09) in ΔHbO_2_ (AN: 4.4 ± 2.9 AΗ: −5.1 ± 3.0 vs. CN: 2.9 ± 2.1 CH: −4 ± 3.1). Also, there was a main group effect in cerebral tissue oxygenation (TSI%) (*p* < 0.05). Furthermore, cerebral TSI was lower in anemic women compared to controls in hypoxia (AH: 51.2 ± 4.2% vs. CH: 56.7 ± 4.5%, *p* = 0.001), and the A group exhibited larger drop in TSI from normoxia to hypoxia compared to the C group (A: 11.1 ± 2.8% vs C: 4.9 ± 3.1%, *p* < 0.001). Accordingly, the level of hypoxemia (C_a_O_2_) was strongly related to the level of absolute cerebral TSI (*r* = 0.95 and *R*^2^ = 0.90) (Fig. 3b). Moreover, cerebral ΔHHb values exhibited larger increase in A group compared to C group when transitioning from normoxia to hypoxia. A condition effect was found for cerebral O_2_Hb (p < 0.001). A trend for interaction (*p* = 0.06) and a condition effect (*p* < 0.001) for cerebral tHb were also found for the A group (N: 8.3 ± 3.3 H: 0.3 ± 5.0) and the C group (N: 5.9 ± 4.4 H: 1.7 ± 2.3).

## Discussion

The present study aimed to investigate the cardiorespiratory response and tissue oxygenation of mildly anemic women during whole-body exercise to exhaustion under normoxia and hypoxia, compared to controls. During maximal exercise on a cycle ergometer, the anemic women compared to controls demonstrated a greater hypoxic reduction of V̇O_2max_ (16.8% vs. 11.8%) concomitant to a reduced cerebral oxygenation. Additionally, when hypoxia was applied, the anemic women did not exhibit further increases in muscle ΔHHb which indicates inability of O_2_ utilization. These responses were also accompanied with lower maximal mean arterial pressure and total peripheral resistance values for the anemic women in hypoxia.

It is well documented that acute lowering of C_a_O_2_ by virtue of [Hb] or FIO_2_ manipulation can alter cardiorespiratory responses and exercise capacity (Amann and Calbet 2008; Amann et al. 2006b; Joyner and Casey 2014; Koskolou et al. 1997a, 1997b). However, most studies have focused on investigating the independent effect of either [Hb] or FIO_2_. Only a few data exist regarding the physiological responses under severe hypoxemia, where both [Hb] and FIO_2_ are reduced (Koskolou et al. 2023; Roach et al. 1999). In our study, SpO_2_ was not different between groups at maximal exercise in both conditions. The hypoxic SpO_2_ values (~ 85%) correspond to moderate level of hypoxemia (Amann et al. 2007). At maximal exercise, *V̇*_E_ did not differ between groups and conditions; however, hypoxia seems to have modified the exercise ventilatory responsiveness of the anemic group who demonstrated increased *V̇*_E_/V̇O_2_ and *V̇*_E_/V̇CO_2_, and attenuated P_ET_CO_2_. Similar respiratory responses were observed in a previous study (Koskolou et al. 2023) which also recorded a higher lactate production from normoxia to hypoxia in the anemic participants, presumably leading to an increased stimulation of peripheral chemoreceptors, as hemoglobin has an important role on blood’s buffering capacity (Mairbaurl 2013; Koskolou et al. 1997b). In the study by Koskolou et al. (2023), the anemic participants suffered more profound hypoxic drop in V̇O_2max_ than the control group (A: 19% vs. C: 10%). Yet, in the present study, the drop of V̇O_2max_ from normoxia to hypoxia had a narrower but still significant difference between the two groups (A: 16.8% vs. C: 11.8%). The higher drop in the study of Koskolou and colleagues (2023) could be explained by the fact that despite the marked differences in [Hb], the V̇O_2max_ values were not significantly different between anemic and controls, suggesting that the physical fitness level of anemic women was high and the hypoxia might have had greater impact on their physiological responses compared to controls (Bartsch and Saltin 2008).

Interestingly, no other study has reported cardiovascular responses during maximal whole-body exercise under additive hypoxemia (anemia + hypoxia). Previous studies that applied the current experimental approach of hypoxemia either used smaller muscle mass exercise (2-leg extension) or they did not record MAP and *Q* (Roach et al. 1999; Koskolou et al. 2023). It has been shown that the cardiovascular system can compensate for reduced arterial oxygen content during submaximal whole-body exercise or when a small mass is engaged (Roach et al. 1999; Koskolou et al. 2023). The amount of muscle mass engaged during exercise in hypoxia can alter substantially the cardiovascular responses during exercise taxing the pumping capacity of the heart (Calbet et al. 2009). In the present study, the anemic group displayed lower maximal mean arterial pressure at the end of hypoxic exercise compared to control group due to a reduced peripheral resistance. This is the first study that indicated a significant reduction of MAP in anemia + hypoxia condition during whole-body exercise, while a previous study (Roach et al. 1999) using two-legged exercise reported a 10% drop of MAP in anemia + hypoxia which did not reach statistical significance. The observed reduction in MAP, in light of no difference in maximal *Q* and HR between groups and conditions, implies that the fall of MAP in the anemic group occurred as a result of peripheral vasodilation due to lower C_a_O_2_ in hypoxia or by changes in whole-blood viscosity (Saltin et al. 1998; Joyner and Casey 2014; Murray et al. 1969). This finding is also in line with studies showing that the underlying mechanism of increased peripheral vasodilation is through ATP and S-nitrosohaemoglobin (SNO-Hb) release mediated by erythrocytes, especially when exposed to hypoxic conditions, contributing to local regulation of blood flow and oxygen delivery (Gonzalez-Alonso et al. 2002, 2006; Ellsworth et al. 1995).

Regarding tissue oxygenation responses, the oxygen diffusion from blood to skeletal muscles depends on the partial pressure gradient, which is already low in hypoxia (Calbet and Lundby 2009). This may lead to a greater diffusive limitation and could be a limiting factor of V̇O_2max_ during whole-body maximal exercise in hypoxic conditions (Wagner 1993). Additionally, iron-deficiency anemia can impose a further challenge to periphery during exercise in hypoxic conditions (Roach et al. 1999; Koskolou et al. 2023). This is the first study that used NIRS-derived measurements to examine the oxygenation responses of skeletal muscle in additive hypoxemic conditions. The anemic women could not increase ΔHHb to support the demands of maximal exercise in hypoxia compared to normoxia, as controls did. SpO_2_ and P_ET_O_2_ values in anemic participants were similar compared to controls, which suggests that the low [Hb] could impair not only oxygen delivery but also diffusion, potentially due to a reduced capillary hematocrit and thus a smaller functional surface area for diffusion (Poole et al. 2022)**.** Another plausible explanation is the low concentration of iron containing enzymes (i.e., cytochrome c oxidase, succinate dehydrogenase) and myoglobin within the muscles that plays a key role in oxygen utilization (Beard 2001). Accordingly, two possible compensatory mechanisms may account for this; the cardiovascular system promoted peripheral vasodilation, as indicated by the drop of total peripheral resistance, to overcome the convective and diffusive limitation of O_2_ utilization from skeletal muscle (Joyner and Casey 2014) or additionally, whole-blood viscosity could also be a contributing factor to the lowering of total peripheral resistance (Murray et al. 1969). The anemic women suffered greater V̇O_2max_ reduction in hypoxia, since there was no change in ΔHHb.

Evidently, arterial hypoxemia contributes to reduced cerebral oxygenation and increased levels of fatigue during exercise (Amann and Kayser 2009; Goodall et al. 2010). C_a_O_2_ acts as the primary regulator of cerebral vasodilation in hypoxia (Hoiland et al. 2016). Despite these known relationships, the effects of anemia and hypoxia interplay on cerebral oxygenation during maximal whole-body exercise remains largely unexplored. In this study, it was shown that cerebral oxygenation was related to C_a_O_2_ (*r* = 0.95); the anemic group exhibited a greater and significant decrease in cerebral tissue oxygenation compared to controls during maximal exercise, and even more so when exposed to hypoxia. Prior studies have shown that the brain can compensate for reduced C_a_O_2_ by increasing O_2_ utilization or improving O_2_ delivery by increasing vascular conductance (Gonzalez-Alonso et al. 2004). Interestingly, reduced cerebral oxygenation has been demonstrated at rest in certain cases of anemia (Sprick et al. 2020). In our study, the anemic women showed profound hyperventilation under hypoxia, unlike controls (Table 2), according to the higher O_2_ and CO_2_ equivalents and the lower P_ET_CO_2_, which affected cerebral oxygenation (Verges et al. 2012). The exercise-induced hyperventilation under hypoxia, possibly overrode cerebral blood flow increases (Verges et al. 2012) and, therefore, no group effect was detected in ΔtHb, which is an index of regional blood volume. Unfortunately, in our study, there was no measurement of cerebral blood flow. Further research is needed to fully elucidate the effects of combining anemia and hypoxia on cerebral oxygenation and cerebral blood flow, particularly in relation to varying levels of skeletal muscle mass engagement during exercise.

When maximal vasodilation occurs during whole-body exercise, mean arterial pressure has to increase to enhance O_2_ delivery (Calbet et al. 2004). In the anemic group exercising under hypoxia, the oxygen delivery was not enhanced due to lower mean arterial pressure response, because cardiac output was not increased leading to cessation of exercise. Therefore, linking the cardiovascular system with tissue oxygenation, as a consequence of the lower mean arterial pressure and the reduced P_ET_CO_2_ in the anemic participants at the end of hypoxic exercise, the cerebral oxygenation was eventually jeopardized in a linear manner with C_a_O_2_ reduction (Fig. 3B) (Hoiland et al. 2016; Imray et al. 2005). Additionally, the reduced cerebral oxygenation had important implication on exercise performance as indicated by the greater reduction of peak power output by ~ 5% (A: 13.1% vs C: 7.7%) in anemic women during exercise in hypoxia compared to normoxia. This reduction could be attributed to blunted central command, through corticospinal inhibition of motor drive resulting in exercise intolerance (Millet et al. 2012; Goodall et al. 2012).

### Perspective

This study adds novel data regarding the cardiovascular response and tissue oxygenation under the current experimental model of additive systemic hypoxemia (anemia + hypoxia) during maximal whole-body exercise on cycle ergometer. The findings of this study revealed that even a moderate level of hypoxia can elicit significant physiological responses, which are not apparent in submaximal exercise (Koskolou et al. 2023), and a greater V̇O_2max_ reduction in chronically anemic women than in controls. These research findings from our lab can have important implications for populations with considerable prevalence of iron-deficiency anemia, such as athletic and military personnel that are often engaged in exercise or operations at moderate altitudes McClung et al. 2006; Burtscher et al. 2024. Also, further research is needed to examine whether cognitive function is compromised and if iron-deficient anemic subjects are more predisposed to acute mountain sickness (AMS). Exploring these parameters is crucial for developing targeted interventions to optimize performance and well-being in this population. This is paramount, since women have higher prevalence of iron deficiency and also appear to have higher risk for AMS (Burtscher et al. 2024; Sim et al. 2019). In addition, sex hormones are known to influence response to hypoxia (Raberin et al. 2024). These factors may have implications for acclimatization to altitude and the overall effectiveness of altitude or hypoxic training in women (Raberin et al. 2024; Govus et al. 2015).

### Limitations

The study was conducted with the minimum sample size (*n* = 8) required based on power analysis. Along with the marginal sample size, the participants were women aged 19–30 with a moderate fitness level, which limits the generalizability of the findings to other age groups, sex, and fitness level. The authors acknowledge that a possible limitation of the study was the lack of cerebral blood flow measurement, restricting the ability to fully interpret cerebrovascular responses and mechanisms. Also, due to technical issues, blood lactate concentrations were not measured in the present study. However, previous work from our laboratory using a similar experimental design reported higher blood lactate (+ 1.68 mmol/L) in the anemic group under hypoxia compared to normoxia (Koskolou et al. 2023), implying that the role of 2,3-DPG in facilitating oxygen delivery to tissues was likely limited during maximal effort in hypoxia, despite its known compensatory function in chronic anemia (Gardner et al. 1977).

## Conclusion

In summary, this study investigated the physiological responses of chronically mild anemic women during maximal whole-body exercise in cycle ergometer under normoxic and hypoxic conditions compared to controls. The anemic women suffered greater V̇O_2max_, peak power output, and cerebral oxygenation reduction in hypoxia compared to controls leading to diminished exercise performance. Also, the anemic group did not exhibit increased skeletal muscle oxygen utilization in hypoxia. They also displayed reduced mean arterial pressure and total peripheral resistance as compensatory mechanisms. These findings indicate that the co-existence of anemia and hypoxia negatively affects cardiorespiratory and tissue oxygenation responses leading to diminished exercise tolerance.

## Data Availability

The data supporting the findings of this study are available from the corresponding author upon reasonable request.
